# Interplay between Carotenoids, Abscisic Acid and Jasmonate Guides the Compatible Rice-*Meloidogyne graminicola* Interaction

**DOI:** 10.3389/fpls.2017.00951

**Published:** 2017-06-08

**Authors:** Tina Kyndt, Kamrun Nahar, Ashley Haeck, Ruben Verbeek, Kristof Demeestere, Godelieve Gheysen

**Affiliations:** ^1^Department of Molecular Biotechnology, Ghent UniversityGhent, Belgium; ^2^Department of Sustainable Organic Chemistry and Technology, Research Group EnVOC, Ghent UniversityGhent, Belgium

**Keywords:** *Oryza sativa*, root knot nematode, hormones, innate immunity, galls

## Abstract

In this study, we have characterized the role of carotenoids and chlorophyll in the compatible interaction between the sedentary root knot nematode (RKN) *Meloidogyne graminicola* and the monocot model plant rice (*Oryza sativa*). Previous transcriptome data showed a differential expression of carotenoid and chlorophyll biosynthesis genes in nematode-induced giant cells and gall tissue. Metabolite measurement showed that galls indeed accumulate chlorophyll a, b and carotenoids, as well as the hormone abscisic acid (ABA). When ABA was externally applied on rice plants, or when ABA-biosynthesis was inhibited, a significant increase in gall formation and nematode development was found, showing the complex role of ABA in this interaction. ABA application suppressed jasmonic acid (JA) levels in the plants, while ABA-biosynthesis inhibition lead to increased JA levels confirming an antagonism between ABA and JA in rice roots. In addition, combined applications of ABA and JA showed that the ABA-effect can overcome JA-induced defense. Based on these observations, we hypothesized that the accumulation of chlorophyll and carotenoid precursors would be beneficial to nematode infection. Indeed, when chemically blocking the carotenoid biosynthesis pathway at different steps, which leads to differential accumulation of carotenoids and chlorophyll in the plants, a positive and clear link between accumulation of carotenoids and chlorophyll and rice susceptibility to RKN was detected.

## Introduction

The root knot nematode (RKN) *Meloidogyne graminicola* is considered to be one of the most damaging biotic causal agents of yield failure in tropical aerobic rice (De Waele and Elsen, 2007; Kreye et al., 2009a,b; Mantelin et al., 2017). In these rice production systems, yield increases of 12–80% have been reported when control measures are applied against RKN (Soriano and Reversat, 2003; Padgham et al., 2004). *M. graminicola* is an obligate, sedentary endoparasite with a wide range of monocot host plants (Mantelin et al., 2017). This RKN transforms some selected root vascular cells into so-called giant cells, which are kept alive as food resource throughout its life cycle (Kyndt et al., 2013). Our research group has published the first comparative transcriptome analysis of rice upon infection with sedentary vs. migratory nematodes, based on mRNA-sequencing of *M. graminicola*-induced galls and *Hirschmanniella oryzae* infected tissue (Kyndt et al., 2012). The results showed that *M. graminicola* is a master in manipulation of the host’s metabolism. Whereas primary metabolic pathways are induced, secondary metabolism is largely suppressed in the gall tissue. Next, we isolated giant cells from the roots and performed mRNA-seq on those specific cells (Ji et al., 2013). These studies have provided important insights into responses of the monocotyledonous rice plant to infection with these root pathogens. One group of genes, found to be strongly activated inside galls induced by RKN in rice roots, is involved in the biosynthesis of photosynthetic pigments, including carotenoids and chlorophyll. Starting from the observation that chloroplast and chlorophyll-biosynthesis genes were induced in giant cells, the presence of chloroplast-like structures was microscopically confirmed inside giant cells (Ji et al., 2013). Noteworthy, these root cells were completely covered from light exposure during these experiments.

**FIGURE 1:**
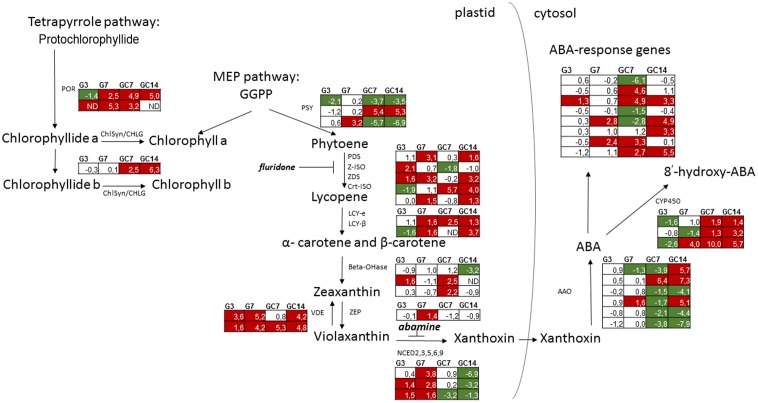
Schematic overview of the expression pattern of genes involved in biosynthesis of chlorophyll, carotenoids and ABA as well as ABA-response in nematode-infected plant tissue. For the different paralogous rice genes annotated to be involved in the different biosynthetic steps the expression pattern (Log2 fold change) is shown in the tables for galls at 3 and 7 dpi (G3 and G7) as well giant cells at 7 and 14 dpi (GC7 and GC14), each time in comparison with the corresponding control material (uninfected root tips or vascular cells, respectively). The gene expression data is derived from Kyndt et al. (2012) for galls and Ji et al. (2013) for giant cells. Significant upregulation is shown in red, while significant downregulation is indicated in green. Annotation of the genes was done using Mapman (Thimm et al., 2004). MEP, methylerythritol 4-phosphate; GGPP, geranylgeranyl diphosphate; POR, protochlorophyllide oxidoreductase; ChlSyn/CHLG, chlorophyll synthase; PSY, phytoene synthase; PDS, phytoene desaturase; Z-ISO, 15-*cis*-zeta-*carotene* isomerase; ZDS, zeta-*carotene* desaturase; Crt-ISO, *cis-trans* isomerase; LCY-e, lycopene cyclase epsilon; LCY-β, lycopene cyclase beta; Beta-OHase, beta hydroxylase; ZEP, zeaxanthin epoxidase; VDE, violaxanthin de-epoxidase; NCED, 9-*cis*-epoxycarotenoid dioxygenases; AAO, abscisic aldehyde oxidase; CYP450, ABA 8′-hydroxylases of the Cytochrome P450 family; ND, no data.

In developing plants, light activates the differentiation of etioplasts into chloroplasts, which is accompanied by biosynthesis of coordinately synthesized carotenoids and chlorophyll pigments. A simplified version of their biosynthesis is shown in **Figure 1**. Chlorophyll can be generated by two distinct biochemical pathways (**Figure 1**), one starting from geranylgeranyl diphosphate (GGPP) generated by the methylerythritol phosphate (MEP) pathway, and the other from protochlorophyllide generated by the tetrapyrrole pathway. Chlorophyll synthase esterifies the hydrophobic hydrocarbon chain of phytyl diphosphate to chlorophyllide, to form chlorophyll a and b (Shalygo et al., 2009).

Biosynthesis of carotenoids also starts from GGPP. The phytoene synthase gene encodes PSY, the first dedicated and rate-limiting enzyme of carotenogenesis. Phytoene is metabolized to lycopene by a linear series of desaturation and isomerization involving four enzymes, among which phytoene desaturase (PDS). Two lycopene cyclases (LCY) then convert lycopene into alpha or beta-carotene, which are subsequently hydroxylated to xanthophylls. Xanthophylls are essential components of the photosynthetic apparatus, and serve as precursors for biosynthesis of abscisic acid (ABA) in chloroplasts and other plastids. ABA biosynthesis starts from the epoxidation of zeaxanthin to violaxanthin, catalyzed by zeaxanthin epoxidase (ZEP) (Agrawal et al., 2001). Then, oxidative cleavage occurs by a family of 9-*cis*-epoxycarotenoid dioxygenases (NCED) leading to the production of xanthoxin, the first cytoplasmic precursor for ABA (Schwartz et al., 2003). In the cytosol, xanthoxin is converted to abscisic aldehyde by a short-chain dehydrogenase reductase (Cheng et al., 2002), which is then oxidized to ABA by abscisic aldehyde oxidases (AAO, Seo et al., 2004). With high levels of ABA, it starts to catabolize into inactive forms. The major catabolic pathway is hydroxylation by 8′-hydroxylases of the cytochrome P450 gene family (Vallabhaneni and Wurtzel, 2010).

Plants respond to pathogen infection using defense mechanisms, that are known to be activated by the plant hormones salicylic acid (SA), ethylene (ET), and jasmonate (JA) (Grant and Jones, 2009; Robert-Seilaniantz et al., 2011). In contrast to these traditional ‘defense-related’ hormones, the role of ABA in the plant defense response is more complex as it promotes resistance in some plant–pathogen interactions, whereas it increases susceptibility in others (Asselbergh et al., 2008). In addition to the specific role of ABA depending on the interaction, it deserves to be noted that most scientific literature about the role of ABA in plant defense is based on interactions with shoot pathogens. Belowground pathogenic interactions tend to be neglected, although the belowground defense system is remarkably different from that in aboveground tissues (Rasmann and Agrawal, 2008; Balmer and Mauch-Mani, 2013). For instance, in the maize-*Colletotrichum graminicola* interaction, roots exhibited higher accumulation of SA, JA, and ABA upon infection, and the general pathogen-induced metabolomics profile of roots was shown to be strongly different from shoots (Balmer et al., 2013). In plant leaves, the ABA–SA reciprocal antagonism has been well-documented (Asselbergh et al., 2008; Yasuda et al., 2008; Ton et al., 2009; Cao et al., 2011). In rice, for instance, ABA suppresses SA-induced expression of *OsNPR1* and *OsWRKY45*, whereas overexpression of these genes alleviates ABA-induced susceptibility to *M. grisea* (Jiang et al., 2010). Our research group has recently demonstrated that exogenous 50 μM ABA treatment compromised rice defense toward the migratory root nematode *Hirschmanniella oryzae* (Nahar et al., 2012). Gene expression analyses using qRT-PCR demonstrated that this disease inducing effect of ABA is likely to be due to an antagonistic interaction between ABA and the SA/JA/ET-based root defense system, among which jasmonate appears to play a key role (Nahar et al., 2011).

In this paper, we present an investigation of the role of photosynthetic pigments and ABA in the compatible interaction between rice and the sedentary RKN *M. graminicola*. To this end, we investigated (1) chlorophyll, carotenoid and ABA levels in galls at different time points after inoculation in comparison with uninfected roots, and (2) the effect of ABA-treatment, and different inhibitors on chlorophyll/carotenoid levels and the root hormone balance in relation to the development of galls and nematodes in the rice roots.

## Materials and Methods

### Plant Material and Growth Conditions

Rice (*Oryza sativa*) seeds of cultivar Nipponbare were provided by the United States Department of Agriculture (GSOR-100). Seeds of *PBZ1*::*OsAOS2* rice plants (Mei et al., 2006) were kindly provided by Dr. Yinong Yang (Penn State University, United States). Seeds were germinated on wet filter paper placed in the petri dish for 4 days at 30°C prior to transplantation in the specially made polyvinyl-chloride (PVC) tubes containing a mixture of fine sand and synthetic absorbent polymer (SAP) substrate (Reversat et al., 1999). The polymer used is Aquaperla^®^ (DCM, Belgium). Each PVC tube contained one plant. The plants were further kept in the plant room at 26°C, 12 h/12 h light regime (150 μmol/m^2^s) and relative humidity of 70–75%. The plants were maintained by supplying Hoagland solution as a source of nutrients at the rate of 10 ml three times a week.

### Infection Experiments

*Meloidogyne graminicola* was originally isolated in the Philippines (Batangas) and was kindly provided by Dirk De Waele (Catholic University, Leuven, Belgium). A nematode culture was maintained on susceptible rice plants grown in potting soil, under light and temperature conditions as described above. About 3 months after inoculation, infected rice roots were cut into 1 mm pieces and nematodes were extracted using a Baermann funnel. The nematode suspension was collected 48 h later and concentrated by centrifugation for 10 min at 1500 rpm at room temperature. Nematode counts were done under light microscopy to estimate the amount of nematodes in the suspension. Fifteen-day-old rice plants were inoculated with 250 juveniles of *M. graminicola* or mock inoculated with water. The infection level of the plants was evaluated at 14 days after inoculation by counting the number of galls and nematodes per plant. To visualize the galls and nematode development, roots were boiled for 3 min in 0.8% acetic acid and 0.013% acid fuchsin. They were washed with running tap water and then destained in acidified glycerol. The number of egg-laying females (ELFs) is considered as a measure of nematode development and an estimate of reproduction.

### Chemical Treatments

Abscisic acid, fluridone, and methyl jasmonate (MeJA) were purchased from Sigma–Aldrich (Overijse, Belgium) and abamine was purchased from Bio-Connect (Huissen, The Netherlands). All chemicals were dissolved in separate vaporizers in a few drops of methanol prior to diluting in water containing 0.02% (v/v) Tween 20. Intact 17 day-old seedlings were sprayed on the leaves with vaporizers until run off with a fine mist of either compound at the indicated concentrations. Distilled water containing 0.02% (v/v) Tween 20, and a few drops of methanol was used as a control treatment. All experiments were repeated three times independently with similar results. In infection experiments, the chemicals were sprayed 24 h before nematode inoculation.

### Data Collection and Statistical Analyses

All statistical analyses were performed in SPSS. Normality of the data was checked by applying the Kolmogorov–Smirnov test of normality (α = 0.05). Homoscedasticity of the data was checked by applying the Levene test (α = 0.05). If the assumptions of normality and homoscedasticity of the data were found to be fulfilled, ANOVA and Duncan’s multiple range test were applied (α = 0.05). If these assumptions were not fulfilled, a non-parametric Mann–Whitney test was used (α = 0.05).

### RNA Extraction, cDNA Synthesis, and qRT-PCR

Total root RNA was extracted using TRIzol (Invitrogen) following the manufacturer’s instructions. For each treatment, two biological replicates were taken, consisting of a pool of at least four plants. The RNA concentration and purity were measured using the NanoVue spectrophotometer (GE Healthcare). To remove contaminating DNA, the extract was treated with DNaseI. Three microgram of RNA was treated with 1 U of DNaseI (Fermentas). qRT-PCR was performed and analyzed as described in Nahar et al. (2012). Expression levels were normalized using two reference genes, *OsEXP* and *OsEXPNarsai*. Primer pairs are listed in **Supplemantary Table S1**.

### Hormone Measurements

Collected root and shoot material was homogenized using liquid N_2_ and 100 mg of ground material was extracted at -80°C using the modified Bieleski solvent. After filtration and evaporation, chromatographic separation was performed on a U-HPLC system (Thermo Fisher Scientific) equipped with a Nucleodur C18 column (50 mm × 2 mm; 1.8 μm d_p_) and using a mobile phase gradient consisting of acidified methanol and water. Mass spectrometric analysis was carried out in selected-ion monitoring (SIM) mode with a Q Exactive Orbitrap mass spectrometer (Thermo Fisher Scientific), operating in negative electrospray ionization mode at a resolution of 70,000 full width at half maximum. The detailed procedure will be described elsewhere (Haeck et al., in preparation). For each treatment, five biological replicates, each consisting of a pool of at least three plants, were measured.

### Chlorophyll and Carotenoid Measurements

Chlorophylls a, b, and other carotenoids were extracted from 200 mg of root or shoot tissue by incubation for 72 h in 3 ml 100% dimethylsulfoxide at 65°C. Their concentrations were calculated using an absorbance measurement of the extract at 480, 649, and 665 nm and the equations described in Pompelli et al. (2013). For each treatment, five biological replicates, each consisting of a pool of at least three plants, were measured.

## Results

Data obtained by our published mRNA sequencing studies on nematode-induced giant cells and gall tissues in rice (Kyndt et al., 2012; Ji et al., 2013) were investigated here in detail, with a specific focus on the chlorophyll/carotenoid biosynthesis-related transcripts. The results, shown in **Figure 1**, demonstrate that many genes in this pathway show differential expression in infected tissue vs. uninfected rice cells, both in galls (G3 and G7, for 3 and 7 days after inoculation) and in giant cells (GC7 and GC14, for 7 and 14 days after inoculation). In giant cells, expression of genes encoding protochlorophyllide reductases (POR) and chlorophyll synthases (ChlSyn) is generally activated. In galls, only *POR* is clearly activated at 7 days after inoculation (dai), while it is slightly repressed at 3 dai. The three homologs of phytoene synthase (*PSY*) show divergent patterns. However, genes involved in lycopene biosynthesis are generally activated in both galls and giant cells. Lycopene cyclases are induced from 7 dai on, both in galls and giant cells. For beta-hydroxylases (Beta-OHase), induction is seen in the giant cells at 7 dai, while repression occurs at 14 dai. The two violaxanthin de-epoxidase (VDE) paralogs are both strongly activated in galls and giant cells. In galls, zeaxanthin epoxidase and all homologs coding for the key enzyme in ABA synthesis, 9-*cis*-epoxycarotenoid dioxygenase (NCED), are induced, mainly 7 dai. Among the three *OsNCED*-genes, two genes were also up-regulated at 3 dai. However, these genes are not differentially expressed in giant cells at 7 dai and even strongly suppressed in 14 dai giant cells. The expression pattern of *AAO* is contrasting, depending on the paralogs of this gene family. Genes involved in ABA catabolism showed a decreasing trend in 3 dai galls and contrasting patterns in 7 dai galls, but they are strongly induced within the giant cells. Taken together, these data indicate that most chlorophyll, carotenoid and ABA biosynthesis genes are activated in galls. However, inside giant cells, both chlorophyll and carotenoid biosynthesis genes are induced, whereas ABA biosynthesis genes are repressed and genes encoding catabolic enzymes are highly expressed. Based on these expression data, we assume that all these metabolites accumulate in the galls, whereas carotenoids and chlorophyll – but not ABA- accumulate in giant cells.

**FIGURE 2:**
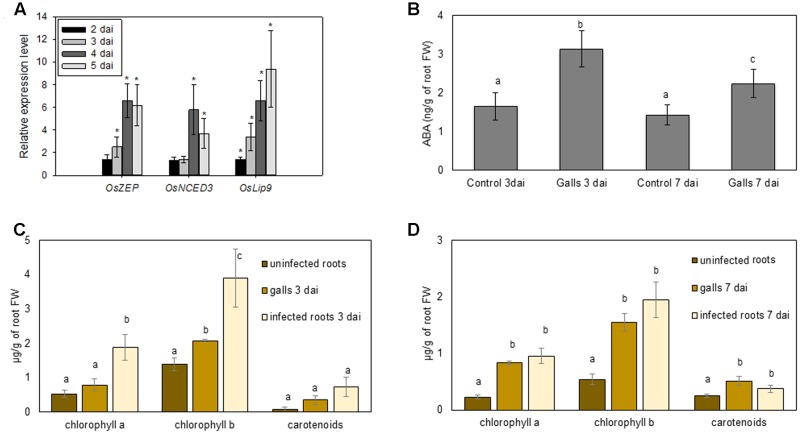
Metabolic analyses and gene expression results of ABA and photosynthetic pigments in nematode-induced galls and root systems in rice. **(A)** qRT-PCR data on three ABA-related genes in *M. graminicola* induced root galls at 2, 3, 4, and 5 days after inoculation (dai). Data are shown as relative expression levels of infected tissue in comparison with control tissue (root tips of uninfected plants, set at expression level of 1). Gene expression levels were normalized using two internal reference genes. Bars represent the mean and standard error of two biological replicates, each containing a pool of three plants. Asterisks indicate statistically significant (*P* < 0.05) different expression in comparison with uninfected plants. **(B)** ABA levels (ng/g of root FW) in galls vs. uninfected root tips (control) at two time points after inoculation, 3 and 7 dai. **(C,D)** chlorophyll a, b and carotenoid levels in uninfected roots, galls and roots neighboring the galls (infected roots) at two time points after inoculation, 3 **(C)** and 7 dai **(D)**. Bars represent the mean and standard error of mean of five pools of three plants. In **(B–D)** different letters indicate significant difference as evaluated using Duncan’s multiple range test (α = 0.05). FW, fresh weight.

In order to independently validate and extend the mRNA-seq observations to more time points, quantitative real-time PCR was used to study gene expression of two biosynthesis and one ABA response marker genes in 2, 3, 4, and 5 dai gall tissue. Results confirmed that *OsZEP* (zeaxanthin epoxidase) was significantly upregulated at all time points of investigation except at 2 dai, whereas *OsNCED3* was induced only at later stages of infection (**Figure 2A**). Expression of the ABA response gene *OsLip9* responded strongly in RKN-induced gall tissue at all time points of observation, with an approximately 10-fold induction by 5 dai relative to non-infected plants (**Figure 2A**).

Although transcriptome data provide indications, only actual measurements can confirm altered metabolite levels in plant tissue. Therefore, internal levels of chlorophyll, carotenoids and ABA were measured in galls at 3 and 7 dai. At both time points, a significant twofold increase of chlorophyll b and ABA levels was detected in galls, compared with uninfected control roots (**Figure 2B** through **2D**). Carotenoids and chlorophyll a also significantly accumulated in 7 dai gall tissue (**Figures 2C,D**).

**FIGURE 3:**
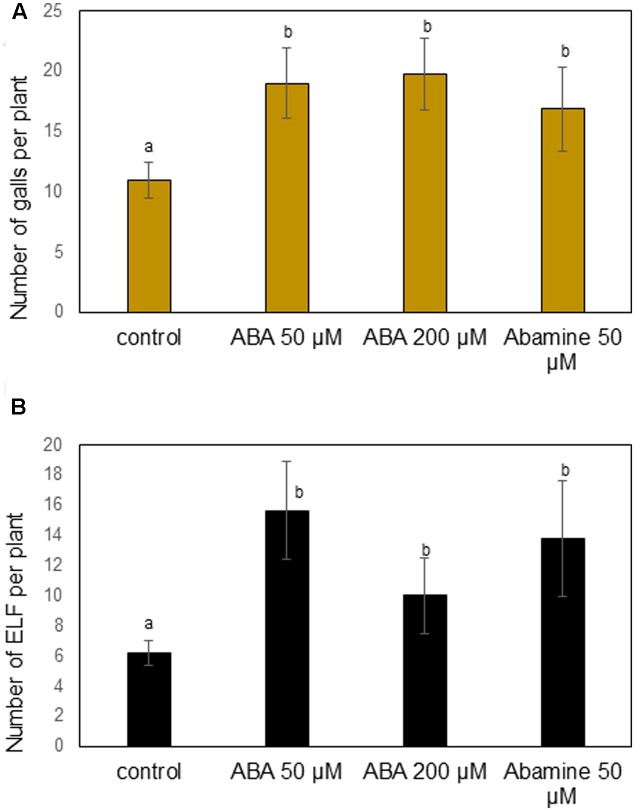
Effect of different concentrations of exogenous abscisic acid (ABA) or ABA-inhibitor (abamine) treatment on rice susceptibility for the root knot nematode *M. graminicola.* At 24 h after chemical treatment, plant roots were inoculated with 250 second stage juveniles of *M. graminicola*. Bars represent the mean (*n* = 8) and standard error of mean as counted at 14 days after inoculation. Different letters indicate statistically significant differences (Duncan’s multiple range test with α = 0.05). Data represent one of three independent experiments with similar results. **(A)** Number of galls per plant and **(B)** number of egg-laying females (ELF) per plant.

Based on our previous research showing that ABA promotes rice susceptibility to *H. oryzae* (Nahar et al., 2012), and given the accumulation of ABA in *M. graminicola*-induced galls, we decided to first focus on the role of ABA in the interaction between rice and RKN. Rice plants were sprayed with two different concentrations of ABA, or NCED-inhibitor abamine, and 1 day later they were inoculated with RKN. The number of galls per plant and nematode development/reproduction were evaluated at 14 dai. The results show that foliar applications of ABA (50 or 200 μM) leads to significantly increased gall formation as compared to the non-treated control (**Figure 3A**). ABA-treated plants also contained an increased total number of nematodes and egg-laying females per plant (**Figure 3B**). Experiments were also done with 100 μM ABA, and showed the same result (data not shown). However, also when applying abamine a significantly increased number of galls and nematodes development was observed (**Figures 3A,B**). These results show a complex role for ABA in the rice-RKN interaction, where both external applications and ABA-biosynthesis inhibition lead to increased susceptibility.

**FIGURE 4:**
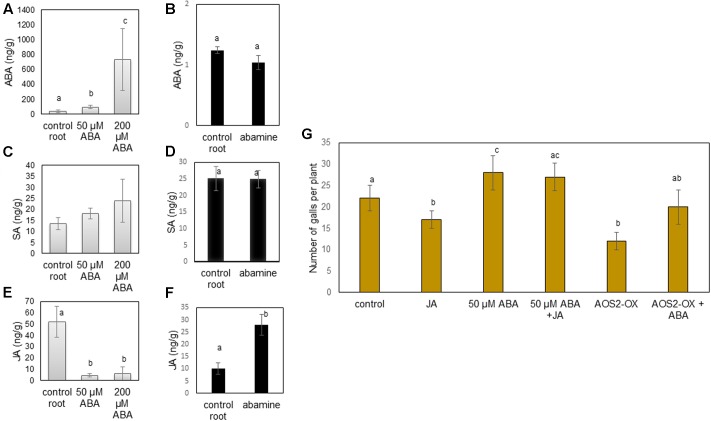
Interaction between ABA and JA in rice roots. **(A–F)** Hormone levels (ng/g of fresh weight) in root tissue of rice plants at 24 h after foliar application of 50 and 200 μM ABA or abamine and corresponding roots of water-treated control plants. **(A,B)** ABA levels **(C,D)** SA levels **(E,F)** JA levels. Bars show the mean and standard error of mean of five biological replicates. **(G)** Effect of application of exogenous JA (MeJA, 100 μM), ABA (50 μM) or both, as well as *PBX::AOS2*-overexpression (AOS2-OX), alone or in combination with ABA (50 μM) on rice susceptibility for the root knot nematode *M. graminicola.* At 24 h after foliar chemical treatment, plant roots were inoculated with 250 second stage juveniles of *M. graminicola*. Bars represent the mean (*n* = 8) and standard error of mean as counted at 14 days after inoculation. Data represent one of three independent experiments with similar results. Different letters indicate statistically significant differences (Duncan’s multiple range test with α = 0.05). ABA, abscisic acid; SA, salicylic acid; JA, jasmonic acid; AOS, allene oxide synthase.

We hypothesized that ABA and abamine might be interfering with the SA/JA based defense system in rice roots. Therefore, SA and JA levels were measured inside roots (**Figure 4A** through **4F**) and shoots (**Supplementary Figure S1**) of abamine or ABA-treated plants at 24 h after foliar application, which is the exact moment when nematodes are usually inoculated (although in this particular experiment the plants were not infected). Results presented in **Figure 4A** show that foliar application of ABA resulted in increased ABA levels in the roots at 24 h after treatment. However, no change in ABA levels was observed when ABA biosynthesis inhibitor abamine was used (**Figure 4B**). Levels of SA were not significantly affected in the roots of both ABA and abamine treated plants (**Figures 4C,D**). Only with 50 μM ABA application, the shoot SA level significantly dropped (**Supplementary Figure S1**). JA levels significantly decreased in both roots and shoots upon ABA-treatment, whereas the JA-level significantly increased upon abamine application (**Figures 4E,F**). These observations show a clear negative correlation between JA and ABA levels in rice roots.

Since JA activation is generally correlated with lower rice susceptibility to RKN (Nahar et al., 2011), and exogenous ABA suppresses internal JA levels at all applied concentrations, we investigated if the ABA-induced susceptibility can be alleviated by enhancing JA levels in the plants, through external application of MeJA or through pathogen-inducible allene oxide synthase 2 (*PBZ::AOS2*) overexpression (AOS2-OX; Mei et al., 2006). Confirming the previously described role of the JA pathway in defense against RKN in rice, the AOS2-OX plants were significantly less susceptible to RKN infection than wild type plants. When combining MeJA with 50 μM ABA–supply or when applying 50 μM on the AOS2-OX line, the ABA-induced susceptibility was not alleviated (**Figure 4G**). On AOS2-OX or upon MeJA application, ABA restored rice susceptibility toward *M. graminicola* to the level of control wild type plants. This indicates that exogenous ABA can overcome JA-induced defense against *M. graminicola* in rice.

**FIGURE 5:**
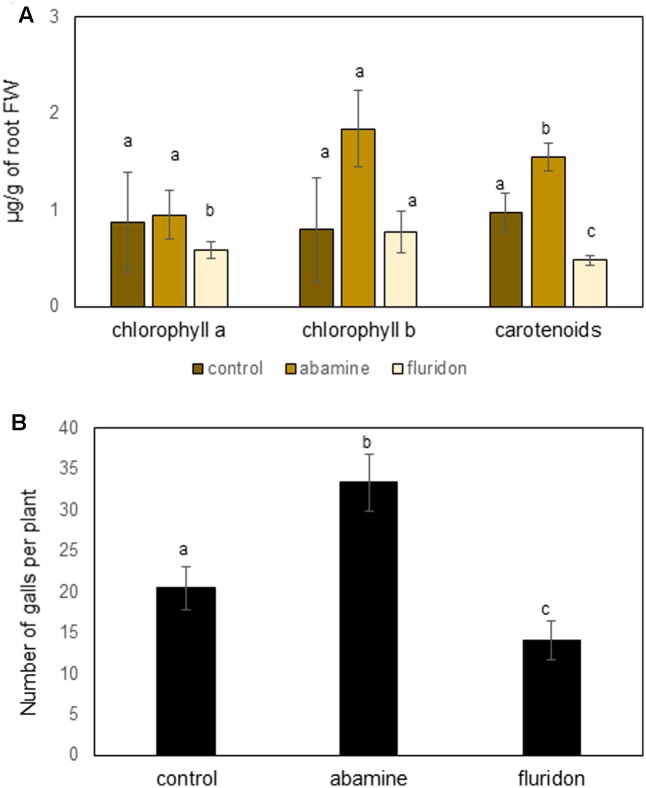
Effect of chemical inhibitors fluridone and abamine on root chlorophyll and carotenoid levels as well as nematode susceptibility. **(A)** Chlorophyll a, b and carotenoid levels in roots of fluridone or abamine treated plants vs. control plants. Plants were treated twice with the inhibitor at 14 and 18 days old, and were sampled at 21-days old. Bars represent the mean and standard error of mean of five pools of three plants. **(B)** Effect of application of fluridone or abamine on rice susceptibility for the root knot nematode *M. graminicola.* At 24 h after foliar chemical treatment, plant roots were inoculated with 250 second stage juveniles of *M. graminicola*. Bars represent the mean (*n* = 8) and standard error of mean as counted at 14 days after inoculation. Data represent one of three independent experiments with similar results. Different letters indicate significant difference as evaluated using Duncan’s multiple range test (α = 0.05). FW, fresh weight.

Although our data shows that exogenous ABA counteracts JA to induce susceptibility, the results obtained with the ABA-biosynthesis inhibitor abamine seem contradictory (**Figure 3**). In addition, **Figure 1** shows that only the first steps of the carotenoid pathway are induced in galls and giant cells, while the genes involved in steps toward biosynthesis of ABA are repressed strongly inside giant cells. Based on these observations, we hypothesized that accumulation of the carotenoid precursors, and potentially also chlorophyll, inside the feeding sites could be beneficial to the RKN. First, we measured carotenoids and chlorophyll in roots of 50 μM ABA-treated plants, and although only marginally significant (*P* = 0.07), a trend toward increased chlorophyll a and carotenoid levels was observable (**Supplementary Figure S2**). Secondly, the carotenoid inhibitor fluridone, which inhibits *PDS*, was applied on rice plants before inoculation, leading to less carotenoid and chlorophyll a accumulation in the rice roots (**Figure 5A**), and reduced nematode susceptibility (**Figure 5B**). Abamine treatment on the other hand was found to lead to significantly increased carotenoid accumulation in the plants (**Figure 5A**) and strongly enhanced susceptibility of the rice plants (**Figure 5B**). These results show a clear positive link between the accumulation of carotenoids and root-knot nematode susceptibility in rice roots.

## Discussion

**FIGURE 6:**
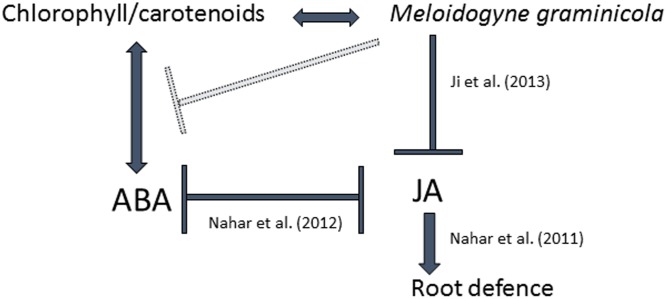
Schematic overview of the results obtained in the current study, in relation to previous results on the jasmonate pathway in the rice-*M. graminicola* interaction. Arrows show positive interactions, while perpendicular lines indicate suppression or antagonism. The here-presented results show that *M. graminicola* infection leads to accumulation of chlorophyll, carotenoids and ABA in infected rice root tissue. We observed that accumulation of these metabolites in rice roots is positively correlated with rice susceptibility to this nematode. Transcriptome data suggests that ABA accumulation is suppressed very locally, inside the giant cells (gray perpendicular line). ABA-treated plants contain significantly less JA, while abamine-treated plants contain more JA, indicating an antagonistic effect of ABA levels on JA biosynthesis in rice roots. Previous studies from our research group indicated this already with gene expression data and further showed that JA is also negatively affecting the ABA-pathway (Nahar et al., 2012). Moreover, the JA pathway was shown to be important for plant defense against this nematode (Nahar et al., 2011) and expression of JA-related genes is suppressed inside giant cells (Ji et al., 2013). ABA, abscisic acid; JA, jasmonic acid.

The sedentary RKN establishes an intimate biotrophic relationship with a host plant through induction of a permanent feeding site inside the root vascular tissue (Gheysen and Mitchum, 2011). In an operational nematode feeding site, the host’s primary metabolic pathways are generally activated, while secondary metabolism is suppressed (Kyndt et al., 2013). Our previously published mRNA-seq data provided evidence for a transcriptional reprogramming of the biosynthesis pathways leading to photosynthetic pigments in galls and giant cells (Kyndt et al., 2012; Ji et al., 2013; **Figure 1**) as well as the formation of chloroplast-like structures in giant cells (Ji et al., 2013). The importance of these pathways were here investigated in more detail with a focus on their interaction with hormonal balances and more specifically the carotenoid-derived hormone ABA. To summarize, a model based on results provided by the here-described experiments is shown in **Figure 6**.

Metabolic measurements on galls confirmed that carotenoids, chlorophyll, and ABA accumulate in these nematode-induced root organs. The accumulation of ABA in plant response to drought stress is well-known (Zhu, 2002), also in monocots (Ye et al., 2011). However, when focusing on biotic stress, the role of ABA is complex and often contradictory. For instance, 4, 20, and 100 μM foliar application of ABA on rice shoots led to a more severe disease response to the rice blast fungus *Magnaporthe oryzae* (Koga et al., 2004), also against an avirulent strain (Jiang et al., 2010), and ABA also negatively influences tomato immunity against *Botrytis cinerea* (Audenaert et al., 2002). On the contrary, ABA treatment (100 μM) induced rice defense against the necrotrophic brown spot pathogen *Cochliobolus miyabeanus* through MAP kinase-mediated repression of ethylene signaling (De Vleesschauwer et al., 2010). Concerning plant-nematode interactions, Karimi et al. (1995) found a lower reproduction rate of *M. incognita* on 100 μM ABA treated potato plants, whereas Nahar et al. (2012) found an increased number of migratory nematodes *H. oryzae* in 50 μM ABA treated rice plants. Taken together, the role of ABA seems plant-pathogen interaction specific (Asselbergh et al., 2008). Our results confirm a complex role for ABA in the here-investigated rice-RKN interaction. ABA foliar supply, which leads to enhanced ABA levels in the shoot and root system, is leading to enhanced susceptibility for RKN. However, also chemical inhibition of ABA-biosynthesis/signaling hampers rice defense against this pathogen. When looking deeper into the transcriptome data of isolated giant cells (Ji et al., 2013; **Figure 1**), we observed that carotenoids and chlorophyll, but not ABA, could be accumulating inside giant cells. Genes involved in the biosynthesis of lycopene and carotenes are generally activated there, whereas genes further downstream, like those encoding NCEDs and AAOs are locally suppressed. Also, ABA-catabolism genes are activated inside giant cells (**Figure 1**). Since hormone measurements show that ABA does accumulate in galls (**Figure 2B**), we hypothesize that ABA accumulates in the neighboring tissues (cells next to the giant cells), probably as a root defense response upon the invading parasite. Although isolation of giant cells for hormone measurement could provide evidence for this statement, collecting the required amount of material to exceed the quantification limits of our technique would be technically very challenging.

Jasmonic acid plays a predominant role in resistance against the biotrophic RKN, while SA has only a minor defense-inducing effect against most parasitic nematodes in rice, tomato, and Arabidopsis (Owen et al., 2002; Nandi et al., 2003; Branch et al., 2004; Nahar et al., 2012; Kammerhofer et al., 2015; Gleason et al., 2016). In both Arabidopsis and tomato, ABA-induced susceptibility against pathogens correlates with repressed SA-related gene expression, suggesting that ABA is able to supress SA biosynthesis (Audenaert et al., 2002; de Torres Zabala et al., 2009) and SA-mediated PTI responses (de Torres Zabala et al., 2009). Our data (**Supplementary Figure S1**) indeed confirm that external ABA application (at 50 μM, but not at 200 μM) negatively affects SA levels in the shoots. However, ABA and SA levels are not negatively correlated in the rice root system (**Figure 4**), confirming previous studies showing that the plant root defense network is strikingly different from the shoot system (reviewed in Balmer and Mauch-Mani, 2013). The here-reported results rather show an inverse correlation between ABA and JA levels in the rice roots (**Figure 4**). Our research group previously demonstrated that ABA-induced susceptibility of rice roots for the migratory nematode *H. oryzae* (Nahar et al., 2012) was correlated with repression of gene expression in the JA- as well as SA- and ET-pathways (Nahar et al., 2012). Here, we extend these observations by demonstrating the antagonism between ABA and JA levels in the roots. While ABA supply leads to reduced JA levels in the roots, abamine treatment enhances JA levels in the plant, most likely be alleviating the ABA–JA antagonism. The inverse correlation between ABA and JA levels was also seen by Kammerhofer et al. (2015) in their study on very early responses of Arabidopsis to cyst nematode infection. During the migratory phase of the nematode infection process, at 24 h after inoculation, JA levels were induced in the root systems whereas ABA was repressed. However, these results are in contrast to the report of Adie et al. (2007), who showed lower resistance against *Pythium irregulare* and reduced JA levels in Arabidopsis ABA-biosynthesis and signaling mutants, as well as that ABA is required for JA-production and defense activation upon *P. irregulare* infection.

It seems that even within the same plant species ABA can play different roles, differences which are not determined by the pathogen lifestyle. For example, ABA protects Arabidopsis from infection by necrotrophs like *Alternaria brassicicola* and *Plectosphaerella cucumerina* (Ton and Mauch-Mani, 2004), but is involved in susceptibility to other necrotrophs like *Fusarium oxysporum* (Anderson et al., 2004). Importantly for the case of the RKN, we know that these parasites modify the plant metabolism to their own benefit, while at the same time suppressing plant defense pathways (Ji et al., 2013). Notably, RKN are masters at suppression of JA-related genes, very locally inside the giant cells (Ji et al., 2013), most likely through secretion of effectors homologous to Mj-FAR1 (Iberkleid et al., 2013).

The fact that ABA application overcomes JA-induced defense shows that the activation of photosynthetic pigment accumulation in infected tissue has an additional JA-independent function. Since mainly the early steps of the carotenoid pathway are induced in giant cells, this led us to hypothesize that ABA precursors – carotenoids – might have a positive role in giant cell and/or nematode development. Indeed, carotenoid measurements on plants treated with exogenous ABA or carotenoid inhibitor revealed a positive correlation between levels of carotenoids and nematode development in rice roots. Carotenoids cannot be synthesized by animals and must hence be ingested from the diet for the subsequent synthesis of essential molecules such as provitamin A, retinal and retinoic acid (Fraser and Bramley, 2004; Tapiero et al., 2004). Although our data showing carotenoid/chlorophyll accumulation in roots upon exogenous ABA treatment are not statistically significant, data from other scientific studies corroborate that ABA application leads to carotenoid and chlorophyll accumulation in different plants (Ivanov et al., 1995; Haisel et al., 2006; Barickman et al., 2014). In plants, carotenoids provide photo-oxidative protection to cells and tissue against the harmful effects of singlet oxygen and lipid radicals and from the chlorophyll triplet (Stahl and Sies, 2003; Dall’Osto et al., 2006). Whether the carotenoids accumulate in galls as a nutrient source for the nematode or to protect the giant cells from oxidative stress remains an open question. The host-dependent feeding of the parasite precludes to experimentally confirm the nutrient source hypothesis in direct feeding assays.

The question remains whether the here-observed accumulation of photosynthetic pigments and carotenoids in the galls is caused by direct effector-based nematode interference with their biosynthesis or rather is a consequence of the oxidative and/or osmotic stress caused by solute accumulation in the feeding sites and reduced water uptake due to vasculature blockage. Indeed, when susceptible cotton plants are infected by *M. incognita*, water flow through the plant is reduced (Kirkpatrick et al., 1991, 1995) and cyst nematode infections cause symptoms reminiscent of physiological drought (Audebert et al., 2000). Atkinson and Urwin (2012) already reported a positive interactive effect of drought stress and *M. graminicola* infection on rice plants, where nematodes ameliorated the severity of the drought stress. The interaction between abiotic and biotic stress in plants has not been well-studied, but seems to follow a non-additive pattern (Atkinson et al., 2013). Less irrigated cotton plants were more susceptible to root galling than irrigated plants (Davis et al., 2014). Taken together, the problems encountered with RKN in aerobic rice systems (De Waele and Elsen, 2007) might be partially due to a higher plant susceptibility related to ABA and carotenoid accumulation.

## Author Contributions

TK, KN, and GG designed the study and the experimental set-up. KN, RV, and TK executed the experiments. AH and KD analyzed hormone levels in the plant tissues. TK wrote the manuscript, with input from all other authors.

## Conflict of Interest Statement

The authors declare that the research was conducted in the absence of any commercial or financial relationships that could be construed as a potential conflict of interest.

## Supplementary Material

FIGURE S1 Hormone levels (ng/g of FW) in shoot tissue of rice plants at 24 h after foliar application of 50 or 200 μM abscisic acid (ABA) vs. shoots of water-treated control plants. **(A)** ABA levels **(B)** salicylic acid (SA) levels **(C)** jasmonic acid (JA) levels. Bars show the mean and standard error of mean of five biological replicates. FW, fresh weight.

FIGURE S2 Chlorophyll a, b and carotenoid levels in roots of 50 μM ABA treated plants vs. control plants at 24 h after foliar chemical treatment. Bars represent the mean and standard error of mean of five pools of three plants. No significant differences were detected. FW, fresh weight.

TABLE S1 Overview of the reference and target genes used in this study, showing their GenBank accession/locus numbers and the primer pair used for qRT-PCR.

## References

[B1] Adie B. A. Perez-Perez J. Perez-Perez M. M. Godoy M. Sanchez-Serrano J. J. Schmelz E. A. (2007). ABA is an essential signal for plant resistance to pathogens affecting JA biosynthesis and the activation of defenses in *Arabidopsis*. *Plant Cell* 19 1665–1681. 10.1105/tpc.106.048041 17513501 PMC1913739

[B2] Agrawal G. K. Yamazaki M. Kobayashi M. Hirochika R. Miyao A. Hirochika H. (2001). Screening of the rice viviparous mutants generated by endogenous retrotransposon *Tos17* insertion. Tagging of a zeaxanthin epoxidase gene and a novel *OsTATC* gene. *Plant Physiol.* 125 1248–1257. 10.1104/pp.125.3.1248 11244106 PMC65605

[B3] Anderson J. P. Badruzsaufari E. Schenk P. M. Manners J. M. Desmond O. J. Ehlert C. (2004). Antagonistic interaction between abscisic acid and jasmonate-ethylene signaling pathways modulates defense gene expression and disease resistance in Arabidopsis. *Plant Cell* 16 3460–3479. 10.1105/tpc.104.025833 15548743 PMC535886

[B4] Asselbergh B. De Vleesschauwer D. Hofte M. (2008). Global switches and fine-tuning - ABA modulates plant pathogen defense. *Mol. Plant Microbe Interact.* 21 709–719. 10.1094/mpmi-21-6-0709 18624635

[B5] Atkinson N. J. Lilley C. J. Urwin P. E. (2013). Identification of genes involved in the response of Arabidopsis to simultaneous biotic and abiotic stresses. *Plant Physiol.* 162 2028–2041. 10.1104/pp.113.222372 23800991 PMC3729780

[B6] Atkinson N. J. Urwin P. E. (2012). The interaction of plant biotic and abiotic stresses: from genes to the field. *J. Exp. Bot.* 63 3523–3543. 10.1093/jxb/ers100 22467407

[B7] Audebert A. Coyne D. L. Dingkuhn M. Plowright R. A. (2000). The influence of cyst nematodes (*Heterodera sacchari*) and drought on water relations and growth of upland rice in Cote d’Ivoire. *Plant Soil* 220 235–242. 10.1023/a:1004734415254

[B8] Audenaert K. De Meyer G. B. Hofte M. M. (2002). Abscisic acid determines basal susceptibility of tomato to *Botrytis cinerea* and suppresses salicylic acid-dependent signaling mechanisms. *Plant Physiol.* 128 491–501. 10.1104/pp.128.2.491 11842153 PMC148912

[B9] Balmer D. de Papajewski D. V. Planchamp C. Glauser G. Mauch-Mani B. (2013). Induced resistance in maize is based on organ-specific defence responses. *Plant J.* 74 213–225. 10.1111/tpj.12114 23302050

[B10] Balmer D. Mauch-Mani B. (2013). More beneath the surface? Root versus shoot antifungal plant defenses. *Front. Plant Sci.* 4:256 10.3389/fpls.2013.00256 PMC3709096 23874350

[B11] Barickman T. Kopsell D. A. Sams C. E. (2014). Abscisic acid increases carotenoid and chlorophyll concentrations in leaves and fruit of two tomato genotypes. *JASHS* 139 261–266.

[B12] Branch C. Hwang C. F. Navarre D. A. Williamson V. M. (2004). Salicylic acid is part of the Mi-1-mediated defense response to root-knot nematode in tomato. *Mol. Plant Microbe Interact.* 17 351–356. 10.1094/mpmi.2004.17.4.351 15077667

[B13] Cao F. Y. Yoshioka K. Desveaux D. (2011). The roles of ABA in plant-pathogen interactions. *J. Plant Res.* 124 489–499. 10.1007/s10265-011-0409-y 21380629

[B14] Cheng W. H. Endo A. Zhou L. Penney J. Chen H. C. Arroyo A. (2002). A unique short-chain dehydrogenase/reductase in Arabidopsis glucose signaling and abscisic acid biosynthesis and functions. *Plant Cell* 14 2723–2743. 10.1105/tpc.006494 12417697 PMC152723

[B15] Dall’Osto L. Lico C. Alric J. Giuliano G. Havaux M. Bassi R. (2006). Lutein is needed for efficient chlorophyll triplet quenching in the major LHCII antenna complex of higher plants and effective photoprotection in vivo under strong light. *BMC Plant Biol.* 6:32 10.1186/1471-2229-6-32 PMC1769499 17192177

[B16] Davis R. F. Earl H. J. Timper P. (2014). Effect of simultaneous water deficit stress and *Meloidogyne incognita* infection on cotton yield and fiber quality. *Phytopathology* 104 30–30. PMC4077171 24987162

[B17] de Torres Zabala M. Bennett M. H. Truman W. H. Grant M. R. (2009). Antagonism between salicylic and abscisic acid reflects early host-pathogen conflict and moulds plant defence responses. *Plant J.* 59 375–386. 10.1111/j.1365-313X.2009.03875.x 19392690

[B18] De Vleesschauwer D. Yang Y. N. Cruz C. V. Hofte M. (2010). Abscisic acid-induced resistance against the brown spot pathogen *Cochliobolus miyabeanus* in rice involves MAP kinase-mediated repression of ethylene signaling. *Plant Physiol.* 152 2036–2052. 10.1104/pp.109.152702 20130100 PMC2850001

[B19] De Waele D. Elsen A. (2007). Challenges in tropical plant nematology. *Annu. Rev. Phytopathol.* 45 457–485. 10.1146/annurev.phyto.45.062806.094438 17489690

[B20] Fraser P. D. Bramley P. M. (2004). The biosynthesis and nutritional uses of carotenoids. *Prog. Lipid Res.* 43 228–265. 10.1016/j.plipres.2003.10.002 15003396

[B21] Gleason C. Leelarasamee N. Meldau D. Feussner I. (2016). OPDA has key role in regulating plant susceptibility to the root-knot nematode *Meloidogyne hapla* in *Arabidopsis*. *Front. Plant Sci.* 7:1565 10.3389/fpls.2016.01565 PMC5075541 27822219

[B22] Gheysen G. Mitchum M. G. (2011). How nematodes manipulate plant development pathways for infection. *Curr. Opin. Plant Biol.* 14 415–421. 10.1016/j.pbi.2011.03.012 21458361

[B23] Grant M. R. Jones J. D. G. (2009). Hormone (dis)harmony moulds plant health and disease. *Science* 324 750–752. 10.1126/science.1173771 19423816

[B24] Haisel D. Pospišilová J. Synková H. Schnablová R. Bat’ková P. (2006). Effects of abscisic acid or benzyladenine on pigment contents, chlorophyll fluorescence, and chloroplast ultrastructure during water stress and after rehydration. *Photosynthetica* 44 606–614. 10.1007/s11099-006-0079-5

[B25] Iberkleid I. Vieira P. Engler J. D. Firester K. Spiegel Y. Horowitz S. B. (2013). Fatty acid-and retinol-binding protein, Mj-FAR-1 induces tomato host susceptibility to root-knot nematodes. *PLoS ONE* 8:e64586 10.1371/journal.pone.0064586 PMC3661543 23717636

[B26] Ivanov A. G. Krol M. Maxwell D. Huner N. P. A. (1995). Abscisic acid induced protection against photoinhibition of PSII correlates with enhanced activity of the xanthophyll cycle. *FEBS Lett.* 371 61–64. 10.1016/0014-5793(95)00872-7 7664885

[B27] Ji H. L. Gheysen G. Denil S. Lindsey K. Topping J. F. Nahar K. (2013). Transcriptional analysis through RNA sequencing of giant cells induced by *Meloidogyne graminicola* in rice roots. *J. Exp. Bot.* 64 3885–3898. 10.1093/jxb/ert219 23881398 PMC3745741

[B28] Jiang C. J. Shimono M. Sugano S. Kojima M. Yazawa K. Yoshida R. (2010). Abscisic acid interacts antagonistically with salicylic acid signaling pathway in rice-*Magnaporthe grisea* interaction. *Mol. Plant Microbe Interact.* 23 791–798. 10.1094/mpmi-23-6-0791 20459318

[B29] Kammerhofer N. Radakovic Z. Regis J. M. A. Dobrev P. Vankova R. Grundler F. M. W. (2015). Role of stress-related hormones in plant defence during early infection of the cyst nematode *Heterodera schachtii* in Arabidopsis. *New Phytol.* 207 778–789. 10.1111/nph.13395 25825039 PMC4657489

[B30] Karimi M. Van Montagu M. Gheysen G. (1995). Exogenous application of abscisic acid to potato plants suppresses reproduction of *Meloidogyne incognita*. *Meded. Fac. Landbouwkd. Toegep. Biol. Wet. Univ. Gent* 60 1033–1035.

[B31] Kirkpatrick T. L. Oosterhuis D. M. Wullschleger S. D. (1991). Interaction of *Meloidogyne incognita* and water stress in two cotton cultivars. *J. Nematol.* 23 462–467. 19283156 PMC2619190

[B32] Kirkpatrick T. L. vanIersel M. W. Oosterhuis D. M. (1995). Influence of *Meloidogyne incognita* on the water relations of cotton grown in microplots. *J. Nematol.* 27 465–471. 19277313 PMC2619637

[B33] Koga H. Dohi K. Mori M. (2004). Abscisic acid and low temperatures suppress the whole plant-specific resistance reaction of rice plants to the infection of *Magnaporthe grisea*. *Physiol. Mol. Plant Pathol.* 65 3–9. 10.1016/j.pmpp.2004.11.002

[B34] Kreye C. Bouman B. A. M. Castaneda A. R. Lampayan R. M. Faronilo J. E. Lactaoen A. T. (2009a). Possible causes of yield failure in tropical aerobic rice. *Field Crops Res.* 111 197–206. 10.1016/j.fcr.2008.12.007

[B35] Kreye C. Bouman B. A. M. Reversat G. Fernandez L. Cruz C. V. Elazegui F. (2009b). Biotic and abiotic causes of yield failure in tropical aerobic rice. *Field Crops Res.* 112 97–106. 10.1016/j.fcr.2009.02.005

[B36] Kyndt T. Denil S. Haegeman A. Trooskens G. Bauters L. Van Criekinge W. (2012). Transcriptional reprogramming by root knot and migratory nematode infection in rice. *New Phytol.* 196 887–900. 10.1111/j.1469-8137.2012.04311.x 22985291

[B37] Kyndt T. Vieira P. Gheysen G. de Almeida-Engler J. (2013). Nematode feeding sites: unique organs in plant roots. *Planta* 238 807–818. 10.1007/s00425-013-1923-z 23824525

[B38] Mantelin S. Bellafiore S. Kyndt T. (2017). *Meloidogyne graminicola*: a major threat to rice agriculture. *Mol. Plant Pathol.* 18 3–15. 10.1111/mpp.12394 26950515 PMC6638252

[B39] Mei C. S. Qi M. Sheng G. Y. Yang Y. N. (2006). Inducible overexpression of a rice allene oxide synthase gene increases the endogenous jasmonic acid level, PR gene expression, and host resistance to fungal infection. *Mol. Plant Microbe Interact.* 19 1127–1137. 10.1094/mpmi-19-1127 17022177

[B40] Nahar K. Kyndt T. De Vleesschauwer D. Hofte M. Gheysen G. (2011). The jasmonate pathway is a key player in systemically induced defense against root knot nematodes in rice. *Plant Physiol.* 157 305–316. 10.1104/pp.111.177576 21715672 PMC3165880

[B41] Nahar K. Kyndt T. Nzogela Y. B. Gheysen G. (2012). Abscisic acid interacts antagonistically with classical defense pathways in rice-migratory nematode interaction. *New Phytol.* 196 901–913. 10.1111/j.1469-8137.2012.04310.x 22985247

[B42] Nandi B. Kundu K. Banerjee N. Babu S. P. S. (2003). Salicylic acid-induced suppression of *Meloidogyne incognita* infestation of okra and cowpea. *Nematology* 5 747–752. 10.1163/156854103322746922

[B43] Owen J. Green C. D. Deverall B. J. (2002). A benzothiadiazole applied to foliage reduces development and egg deposition by *Meloidogyne* spp. in glasshouse-grown grapevine roots. *Australas. Plant Pathol.* 31 47–53. 10.1071/ap01068

[B44] Padgham J. L. Duxbury J. M. Mazid A. M. Abawi G. S. Hossain M. (2004). Yield loss caused by *Meloidogyne graminicola* on lowland rainfed rice in Bangladesh. *Journal of Nematology* 36 42–48. 19262786 PMC2620730

[B45] Pompelli F. Franca S. C. Tigre R. C. de Oliveira M. T. Sacilot M. Pereira E. C. (2013). Spectrophotometric determinations of chloroplastidic pigments in acetone, ethanl and dimethylsulphoxide. *Rev. Bras. Biocien.* 11 52–58.

[B46] Rasmann S. Agrawal A. A. (2008). In defense of roots: a research agenda for studying plant resistance to belowground herbivory. *Plant Physiol.* 146 875–880. 10.1104/pp.107.112045 18316643 PMC2259042

[B47] Reversat G. Boyer J. Sannier C. Pando-Bahuon A. (1999). Use of a mixture of sand and water-absorbent synthetic polymer as substrate for the xenic culturing of plant-parasitic nematodes in the laboratory. *Nematology* 1 209–212.

[B48] Robert-Seilaniantz A. Grant M. Jones J. D. G. (2011). Hormone crosstalk in plant disease and defense: more than just JASMONATE-SALICYLATE antagonism. *Annu. Rev. Phytopathol.* 49 317–343. 10.1146/annurev-phyto-073009-114447 21663438

[B49] Schwartz S. H. Tan B. C. McCarty D. R. Welch W. Zeevaart J. A. D. (2003). Substrate specificity and kinetics for VP14 a carotenoid cleavage dioxygenase in the ABA biosynthetic pathway. *Biochim. Biophys. Acta* 1619 9–14. 10.1016/s0304-4165(02)00422-1 12495810

[B50] Seo M. Aoki H. Koiwai H. Kamiya Y. Nambara E. Koshiba T. (2004). Comparative studies on the *Arabidopsis* aldehyde oxidase (AAO) gene family revealed a major role of AAO3 in ABA biosynthesis in seeds. *Plant Cell Physiol.* 45 1694–1703. 10.1093/pcp/pch198 15574845

[B51] Shalygo N. Czarnecki O. Peter E. Grimm B. (2009). Expression of chlorophyll synthase is also involved in feedback-control of chlorophyll biosynthesis. *Plant Mol. Biol.* 71 425–436. 10.1007/s11103-009-9532-8 19680747

[B52] Soriano R. Reversat G. (2003). Management of *Meloidogyne graminicola* and yield of upland rice in South-Luzon, Philippines. *Nematology* 5 879–884. 10.1163/156854103773040781

[B53] Stahl W. Sies H. (2003). Antioxidant activity of carotenoids. *Mol. Aspects Med.* 24 345–351. 10.1016/s0098-2997(03)00030-x 14585305

[B54] Tapiero H. Townsend D. M. Tew K. D. (2004). The role of carotenoids in the prevention of human pathologies. *Biomed. Pharmacother.* 58 100–110. 10.1016/j.biopha.2003.12.006 14992791 PMC6361147

[B55] Thimm O. Bläsing O. Gibon Y. Nagel A. Meyer S. Kruger P. (2004). MAPMAN: a user-driven tool to display genomics data sets onto diagrams of metabolic pathways and other biological processes. *Plant J.* 37 914–939. 10.1111/j.1365-313X.2004.02016.x 14996223

[B56] Ton J. Flors V. Mauch-Mani B. (2009). The multifaceted role of ABA in disease resistance. *Trends Plant Sci.* 14 310–317. 10.1016/j.tplants.2009.03.006 19443266

[B57] Ton J. Mauch-Mani B. (2004). beta-amino-butyric acid-induced resistance against necrotrophic pathogens is based on ABA-dependent priming for callose. *Plant J.* 38 119–130. 10.1111/j.1365-313X.2004.02028.x 15053765

[B58] Vallabhaneni R. Wurtzel E. T. (2010). From epoxycarotenoids to ABA: the role of ABA 8′-hydroxylases in drought-stressed maize roots. *Arch. Biochem. Biophys.* 504 112–117. 10.1016/j.abb.2010.07.005 20637177 PMC2957537

[B59] Yasuda M. Ishikawa A. Jikumaru Y. Seki M. Umezawa T. Asami T. (2008). Antagonistic interaction between systemic acquired resistance and the abscisic acid-mediated abiotic stress response in *Arabidopsis*. *Plant Cell* 20 1678–1692. 10.1105/tpc.107.054296 18586869 PMC2483369

[B60] Ye H. Zhu G. H. Liu Y. G. Li Y. X. Zhang J. H. (2011). ABA controls H_2_O_2_ accumulation through the induction of OsCATB in rice leaves under water stress. *Plant Cell Physiol.* 52 689–698. 10.1093/pcp/pcr028 21398647

[B61] Zhu J. K. (2002). Salt and drought stress signal transduction in plants. *Annu. Rev. Plant Biol.* 53 247–273. 10.1146/annurev.arplant.53.091401.143329 12221975 PMC3128348

