# Supplementary figures and images for: Interplay between Carotenoids, Abscisic Acid and Jasmonate Guides the Compatible Rice-Meloidogyne graminicola Interaction
Source: Front Plant Sci. 2017 Jun 8;8:951. doi: 10.3389/fpls.2017.00951 (PMC5462958; doi:10.3389/fpls.2017.00951)

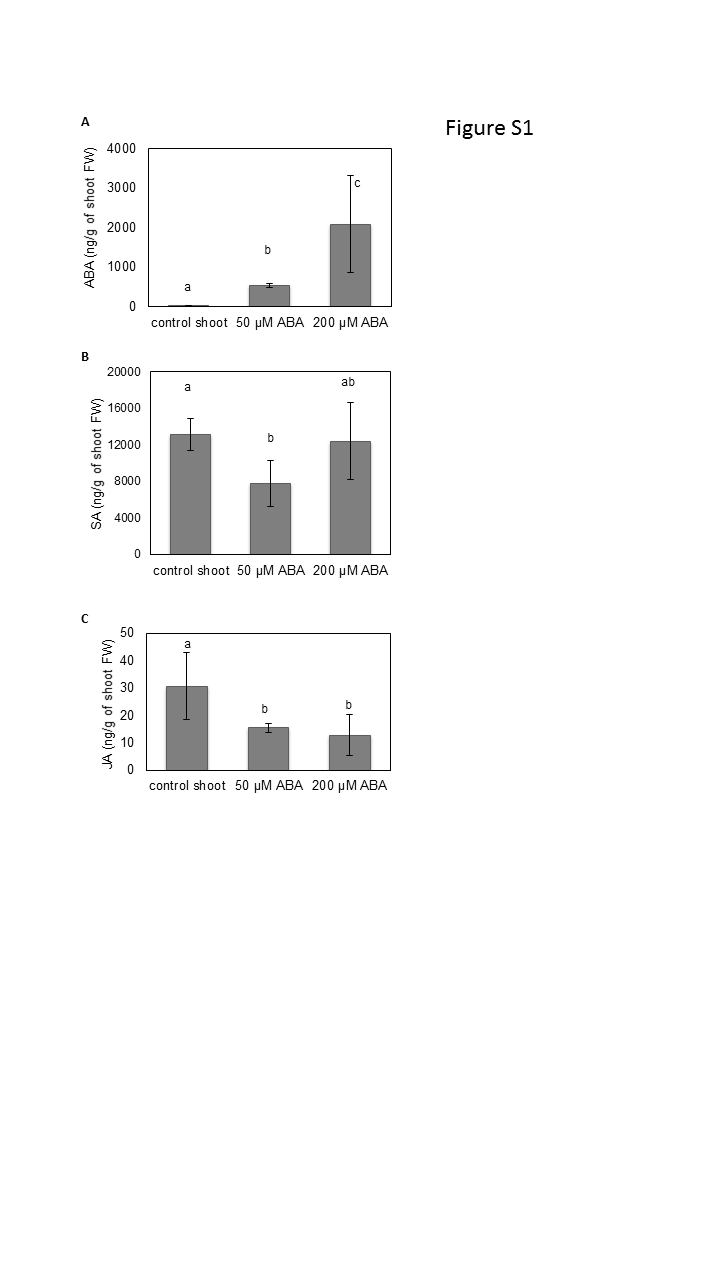

Supplement: FIGURE S1 — Hormone levels (ng/g of FW) in shoot tissue of rice plants at 24 h after foliar application of 50 or 200 μM abscisic acid (ABA) vs. shoots of water-treated control plants. (A) ABA levels (B) salicylic acid (SA) levels (C) jasmonic acid (JA) levels. Bars show the mean and standard error of mean of five biological replicates. FW, fresh weight. [file Image_1.TIF]

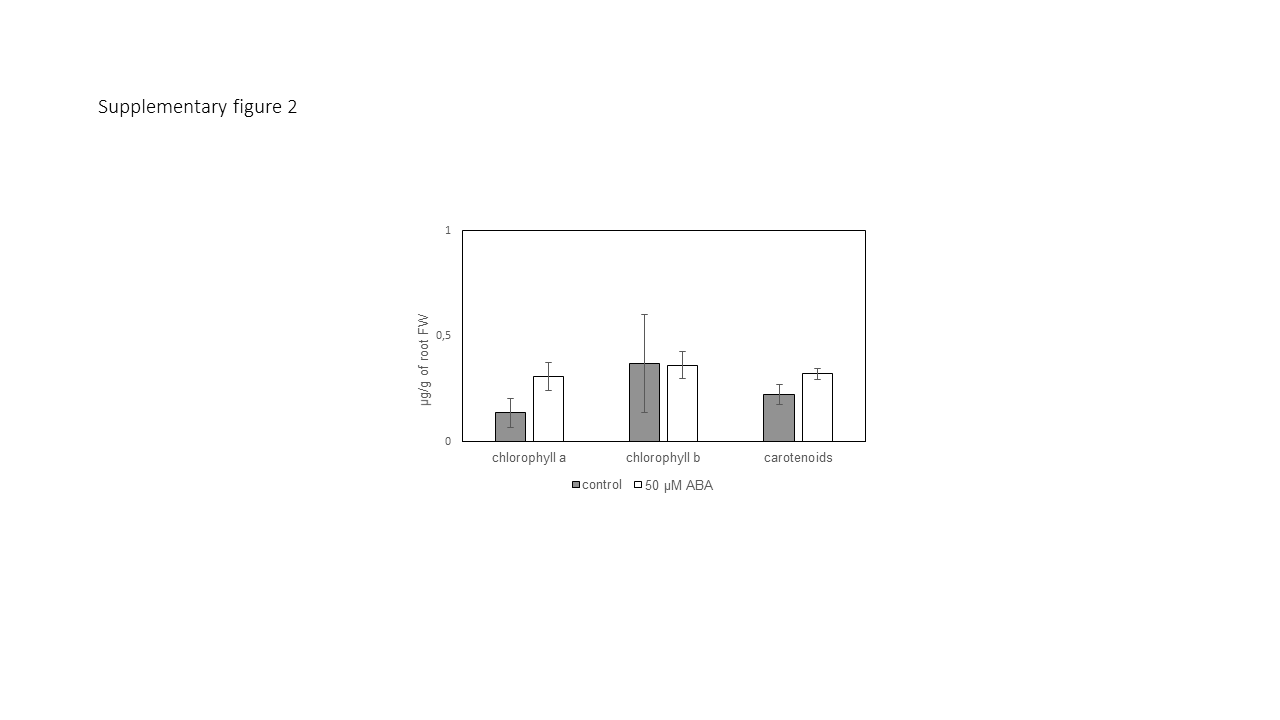

Supplement: FIGURE S2 — Chlorophyll a, b and carotenoid levels in roots of 50 μM ABA treated plants vs. control plants at 24 h after foliar chemical treatment. Bars represent the mean and standard error of mean of five pools of three plants. No significant differences were detected. FW, fresh weight. [file Image_2.TIF]
