# Supplementary material for: Interplay between Carotenoids, Abscisic Acid and Jasmonate Guides the Compatible Rice-Meloidogyne graminicola Interaction
Source: Front Plant Sci. 2017 Jun 8;8:951. doi: 10.3389/fpls.2017.00951 (PMC5462958; doi:10.3389/fpls.2017.00951)
Supplement: TABLE S1 — Overview of the reference and target genes used in this study, showing their GenBank accession/locus numbers and the primer pair used for qRT-PCR. [file Table_1.docx]

**Supplementary table**

Table S1. Overview of the reference and target genes used in this study, showing their GenBank accession/locus numbers and the primer pair used for qRT-PCR

| Reference/Target Gene | GenBank Accession/Locus No. | Forward Primer (5´-3´) | Reverse Primer (5´-3´) |
| --- | --- | --- | --- |
| *OsEXP* (reference gene) | LOC_Os03g27010 | TGTGAGCAGCTTCTCGTTTG | TGTTGTTGCCTGTGAGATCG |
| *OsEXPNarsai* (reference gene) | LOC_Os07g02340 | CACGTTACGGTGACACCTTTT | GACGCTCTCCTTCTTCCTCAG |
| *OsNCED3* (nine-cis-epoxycarotenoid dioxygenase 3) | NM_001057300.2 | GTTCAAGCTCCAGGAGATGC | AGAGGTGGAAGCAGAAGCAG |
| *OsZEP* (zeaxanthin epoxidase | LOC_Os04g37619 | TATGTGACTGATAATGGAAGTG | ATGGCTGACTGAAGTCTCTCGT |
| *OsLip9* (ABA response gene) | AB011367 | CCGGCTACAGAGGAAGTGAG | TCTCCATGATCTTGCCCAGT |
